# Association of Federal Regulations in the United States and Canada With Potential Corneal Donation by Men Who Have Sex With Men

**DOI:** 10.1001/jamaophthalmol.2020.3630

**Published:** 2020-09-24

**Authors:** Michael A. Puente, Jennifer L. Patnaik, Anne M. Lynch, Blake M. Snyder, Chad M. Caplan, Binhan Pham, Helio V. Neves da Silva, Conan Chen, Michael J. Taravella, Alan G. Palestine

**Affiliations:** 1 Department of Ophthalmology, University of Colorado School of Medicine, Aurora; 2 Department of Ophthalmology, Tulane University School of Medicine, New Orleans, Louisiana

## Abstract

**Importance:**

Federal policy in the United States prohibits corneal donation by men who have had sex with another man (MSM) in the preceding 5 years, whereas Canada enforces a 12-month ban. The potential consequences of these policies on corneal donations should be evaluated.

**Objective:**

To estimate the number of potential corneal donations associated with MSM deferral policies in the United States and Canada.

**Design, Setting, and Participants:**

A nonvalidated telephone survey study was conducted of all 65 eye banks in the United States and Canada to investigate how many potential corneal donors were disqualified in 2018 because of federal MSM restrictions. Published demographic data were also used to arrive at a separate estimate. Survey data were gathered from May 2019 to February 2020.

**Main Outcomes and Measures:**

Eye banks were asked if they keep records of referrals disqualified specifically because of the federal MSM restrictions and, if so, how many referrals they disqualified in 2018 owing to MSM status.

**Results:**

Fifty-four of 65 eye banks (83%) responded to the survey, with 30 eye banks reporting they do not keep specific records of MSM deferrals. The remaining 24 eye banks reported disqualifying 360 referrals in 2018 because of MSM status, equating to 720 corneas. The 24 eye banks accounted for 46.2% of corneal donations in the United States and Canada in 2018, yielding an estimate of approximately 1558 corneas rejected that year because of MSM status. A separate estimate using published MSM demographic data indicates that up to 3217 potential corneal donations may have been disqualified in 2018 because of these federal policies.

**Conclusions and Relevance:**

Findings suggest that between 1558 and 3217 corneal donations were disqualified in 2018 because of federal regulations prohibiting corneal donation by men who have had sex with another man in the preceding 5 years in the United States or 1 year in Canada. With modern virologic testing that is reliable within days of HIV exposure and given the global shortage of corneal tissue, these policies should be reevaluated using current scientific evidence to increase the availability of vision-restoring surgery worldwide.

## Key Points

**Question:**

Regarding federal policy in the United States that prohibits corneal donation by men who have had sex with another man (MSM) in the preceding 5 years (or 12 months in Canada), what is the association of these policies with the supply of donated corneas?

**Findings:**

These policies were associated with the disqualification of an estimated 1558 to 3217 corneal donations by otherwise eligible MSM donors in the United States and Canada in 2018.

**Meaning:**

With reliable modern HIV testing and with many countries experiencing severe corneal shortages, these federal policies may be decreasing the availability of vision-restoring surgery, suggesting these policies should be reevaluated in light of current scientific evidence.

## Teaser

This survey study estimates the number of potential corneal donations associated with deferral policies in the United States and Canada for men who have had sex with another man.

## Introduction

Corneal donation involves a rigorous screening process designed to reduce the risk of infectious disease transmission via corneal transplant.^1^ Since the early years of the AIDS pandemic in the 1980s and 1990s, there has been concern for the possibility of HIV transmission through corneal transplants.^2,3,4^ Therefore, on May 20, 1994, the US Public Health Service instituted a policy prohibiting corneal donation by men who have had sex with another man (MSM) in the preceding 5 years.^5^ This regulation continues to be enforced today by the US Food and Drug Administration (FDA).^6^ At the time of the policy’s introduction, HIV screening tests were unreliable up to 6 months after viral exposure,^7^ so it was argued that a categorical exclusion policy for sexually active MSM donors was necessary to protect corneal transplant recipients. However, in the decades since this rule was implemented, HIV testing has become reliable within 4 to 8 days of viral exposure.^8^ With modern virologic testing and a better understanding of the risk of HIV transmission through corneal transplants, the 5-year deferral policy for MSM corneal donors may no longer be supported by current evidence.

There is a well-documented shortage of corneal tissue across the world, with millions of people in need of a corneal transplant.^9,10^ Because the United States exports thousands of corneas annually, any policy that restricts the supply of donated corneas in the United States may reduce the availability of vision-restoring surgery worldwide. Furthermore, this policy impacts not only the potential recipients of corneas from MSM donors but also the families of MSM donors, who are deprived of an opportunity to donate this vision-restoring tissue on behalf of their loved ones.

We aimed to investigate the number of potential corneal donations prevented each year because of MSM deferral policies. Neither the FDA nor the Eye Bank Association of America (EBAA) requires eye banks to keep track of the number of referrals impacted by this rule. To address this issue, our research team (M.A.P., B.M.S., and C.M.C.) individually contacted every eye bank in the United States to arrive at an estimate. Given that the EBAA also certifies all but 1 eye bank in Canada and given that Health Canada also enforces a ban on corneal donation by any man who has had sex with another man in the preceding 12 months,^11^ we contacted every eye bank in Canada as well.

## Methods

### Study Design

For this survey study, a list was obtained of all eye banks in the United States from a database published on the EBAA website.^12^ This database also included all but 1 of the eye banks in Canada, with the exception being the non–EBAA-accredited eye bank of Quebec. We then contacted all 65 of these eye banks (57 in the United States and 8 in Canada, including the eye bank of Quebec) and conducted a nonvalidated retrospective telephone survey asking the following questions: (1) if they document when a referral is disqualified for corneal donation specifically because of MSM contact, (2) how many referrals they rejected in the 2018 calendar year owing to MSM status, and (3) how many corneas were recovered altogether at their eye bank in 2018. For eye banks that said that their initial donor screening was handled by a partner organ procurement organization, we contacted their partner organization to acquire the data. Eye banks that did not respond to our initial inquiry received at least 3 telephone calls over several months before our team ceased inquiring. Survey data were gathered from May 2019 to February 2020. We also obtained a second estimate of MSM donors disqualified because of the FDA and Canadian policies in 2018 using published population-based data on sexual behavior and sexual orientation in the 2 countries^13,14^ and combining this finding with statistics on the number of corneas donated in the 2 countries in 2018.

The Colorado Multiple Institutional Review Board reviewed the protocol for this study and concluded that it did not qualify as human participant research. The study was conducted according to the Declaration of Helsinki.^15^

## Results

Fifty-four of 65 eye banks (83%) responded to our inquiries. Of the 54 eye banks that participated in the study, 24 eye banks (44%) were able to provide a specific number of referrals who were rejected for corneal donation because of the federal MSM deferral policy (Table 1). Thirty eye banks (56%) said such deferrals were only labeled as high risk in their records, being grouped together with other referrals rejected because of intravenous drug use, incarceration, or other behavioral risk factors. Those 30 eye banks reported that it would not be possible to sort which referrals were disqualified specifically because of the MSM policy (Table 2). The remaining 11 eye banks (17%) did not respond to our survey despite receiving at least 3 telephone calls (Table 3).

**Table 1.** List of Eye Banks in the United States and Canada That Provided 2018 Data for Referrals Deferred Specifically Because of Donor Status as Men Who Have Sex With Men

Eye bank	Primary locations
Advancing Sight Network	Birmingham, Alabama
	Huntsville, Alabama
	Memphis, Tennessee
	Mobile, Alabama
	Montgomery, Alabama
Arkansas Lions Eye Bank	Little Rock, Arkansas
SightLife	Seattle, Washington
	Spokane, Washington
	Anchorage, Alaska
	South San Francisco, California
	Irvine, California
	Bethlehem, Pennsylvania
Rocky Mountain Lions Eye Bank	Aurora, Colorado
	Cheyenne, Wyoming
Envision	Boise, Idaho
Kansas Eye Bank & Cornea Research Center	Wichita, Kansas
Baton Rouge Eye Bank	Baton Rouge, Louisiana
Southern Eye Bank	Metairie, Louisiana
Eversight	Ann Arbor, Michigan
	Chicago, Illinois
	Clark, New Jersey
	Cleveland, Ohio
Lions Gift of Sight	Minneapolis, Minnesota
Saving Sight	Columbia, Missouri
	Kansas City, Missouri
	Springfield, Illinois
	Springfield, Missouri
	St Louis, Missouri
New Brunswick Organ and Tissue Program	Saint John, New Brunswick, Canada
Miracles in Sight	Winston-Salem, North Carolina
Lions Eye Bank of Nebraska	Omaha, Nebraska
Regional Tissue Bank	Halifax, Nova Scotia, Canada
Central Ohio Lions Eye Bank	Columbus, Ohio
Oklahoma Lions Eye Bank	Oklahoma City, Oklahoma
Lions VisionGift	Portland, Oregon
Lions Eye Bank of Texas at Baylor College of Medicine	Houston, Texas
	Beaumont, Texas
	Corpus Christi, Texas
	Victoria, Texas
	Harlingen, Texas
Lone Star Lions Eye Bank	Manor, Texas
Western Texas Lions Eye Bank Alliance	San Angelo, Texas
Utah Lions Eye Bank	Murray, Utah
Lions Medical Eye Bank and Research Center of Eastern Virginia	Norfolk, Virginia
Lions Eye Bank of Wisconsin	Madison, Wisconsin

**Table 2.** List of Eye Banks in the United States and Canada That Do Not Track Whether a Referral Is Deferred Specifically Because of Donor Status as Men Who Have Sex With Men

Eye bank	Primary locations
Donor Network of Arizona	Phoenix, Arizona
Eye Bank of British Columbia	Vancouver, British Columbia, Canada
San Diego Eye Bank	San Diego, California
Sierra Donor Services Eye Bank	West Sacramento, California
	Nashville, Tennessee
Lions Eye Institute for Transplant and Research	Tampa, Florida
	Fort Myers, Florida
	Gainesville, Florida
	Orlando, Florida
	Jacksonville, Florida
	Melbourne, Florida
	Pensacola, Florida
	Shreveport, Louisiana
Georgia Eye Bank	Atlanta, Georgia
Hawaii Lions Eye Bank and Makana Foundation	Honolulu, Hawaii
Iowa Lions Eye Bank	Coralville, Iowa
	Des Moines, Iowa
VisionFirst	Indianapolis, Indiana
Kentucky Lions Eye Bank	Louisville, Kentucky
	Lexington, Kentucky
Misericordia Eye Bank	Winnipeg, Manitoba, Canada
CorneaGen/KeraLink	Baltimore, Maryland
	Albuquerque, New Mexico
	San Antonio, Texas
	Waltham, Massachusetts
Mid-America Transplant	St Louis, Missouri
Mississippi Lions Eye Bank	Flowood, Mississippi
LifeShare Carolinas	Charlotte, North Carolina
	Asheville, North Carolina
The Eye-Bank for Sight Restoration	New York, New York
ConnectLife	Williamsville, New York
Cincinnati Eye Bank for Sight Restoration	Cincinnati, Ohio
Lions Eye Bank of West Central Ohio	Dayton, Ohio
Eye Bank of Canada–Ontario Division	Toronto, Ontario, Canada
Lions Eye Bank of Delaware Valley	Philadelphia, Pennsylvania
Lions Eye Bank of Northwest Pennsylvania	Erie, Pennsylvania
Lions Eye Bank of Puerto Rico	San Juan, Puerto Rico
Héma-Québec	Montreal, Quebec, Canada
	Quebec City, Quebec, Canada
Dakota Lions Sight and Health	Sioux Falls, South Dakota
	Rapid City, South Dakota
	Bismarck, North Dakota
	Fargo, North Dakota
Eye Bank of Saskatchewan	Saskatoon, Saskatchewan, Canada
	Regina, Saskatchewan, Canada
East Tennessee Lions Eye Bank	Knoxville, Tennessee
Mid-South Eye Bank for Sight Restoration	Memphis, Tennessee
Great Plains Lions Eye Bank	Lubbock, Texas
Old Dominion Eye Foundation	Richmond, Virginia

**Table 3.** List of Eye Banks in the United States and Canada That Did Not Respond to the Survey

Eye bank	Primary locations
Lions Eye Bank	Calgary, Alberta, Canada
One Legacy	Los Angeles, California
Florida Lions Eye Bank	Miami, Florida
Nevada Donor Network	Las Vegas, Nevada
Central New York Eye and Tissue Bank	Syracuse, New York
Lions Eye Bank at Albany	Albany, New York
Lions Eye Bank for Long Island	Valley Stream, New York
Center for Organ Recovery and Education	Pittsburgh, Pennsylvania
Gift of Life Donor Program Eye Bank	Hershey, Pennsylvania
	Philadelphia, Pennsylvania
Transplant Services Center, UT Southwestern Medical Center	Dallas, Texas
Medical Eye Bank of West Virginia	Charleston, West Virginia

The 24 eye banks that were able to provide specific data reported turning away a total of 360 referrals in 2018 because of MSM sexual activity in the preceding 5 years in the United States or in the preceding 12 months in Canada. Assuming that both eyes would have been recovered in all such donors equates to 720 corneas declined in 1 year. Those 24 eye banks recovered a total of 64 013 corneas in 2018, representing 46.2% of the 138 621 corneas recovered in the United States and Canada that year according to US data from the 2018 EBAA Statistical Report^16^ and Canadian data personally conferred to our team by the EBAA and the eye bank of Quebec (Claudie-Ann Michaud Tremblay, MSc, Héma-Québec, telephone communication, February 24, 2020). Assuming that those eye banks similarly would have accounted for 46.2% of MSM donor deferrals yields an estimate of approximately 1558 (720 ÷ 0.462 = 1558) corneas rejected for consideration for transplant in 2018 because of donor MSM history.

Based on a meta-analysis^13^ estimate that 3.9% of men in the United States have had sexual contact with men (MSM contact) in the preceding 5 years, with a total of 133 576 corneas recovered in the United States in 2018 and with 60% of corneal donors being male,^16^ we calculated that approximately up to 3126 (133 576 × 0.039 × 0.6 = 3126) potential corneal donations would have been disqualified in the United States in 2018 because of the 5-year MSM deferral policy. We were unable to obtain an estimate of the percentage of Canadian men who had engaged in MSM contact in the preceding 12 months, but the 2015 Canadian Community Health Survey found that 3.0% of Canadian men self-identify as either gay or bisexual.^14^ Combining this 3.0% estimate with the total of 5045 corneas recovered in Canada in 2018 according to personal communication from the EBAA (Jennifer Dematteo, MCM, CIC, telephone communication, January 8, 2020) and the eye bank of Quebec (Claudie-Ann Michaud Tremblay, MSc, telephone communication, February 24, 2020), and assuming that 60% of donors were men as in the United States, yields an estimate of approximately 91 corneas that would have been turned away in Canada in 2018 because of the ban on donation by sexually active MSM. Combining these estimates from the United States and Canada suggests that approximately 3217 (3126 + 91 = 3217) corneas were prevented from entering the donor pool in the 2 countries in 2018 because of donor MSM status.

The estimates of 1558 to 3217 potential corneal donations disqualified because of MSM status suggest that the MSM deferral policies were associated with a 1.1% (1558 ÷ [138 621 + 1558] = 1.1%) to 2.3% (3217 ÷ [138 621 + 3217] = 2.3%) reduction in the corneal donor supply in the United States and Canada in 2018. These were estimates for the combined total of the 2 countries.

## Discussion

This study revealed that federal restrictions on corneal donation by MSM were associated with a substantial reduction in the number of corneas donated in the United States and Canada in 2018. The eye banks that were contacted consistently stated that they had never previously reported the number of referrals rejected specifically because of MSM status, making our study the first to date to report the impact of this policy that has been in place since the early years of the AIDS pandemic.

Although the MSM deferral policy was instituted primarily as a means of preventing donors infected with HIV from entering the donor pool, there are now other means of screening out such donors. Current FDA, Health Canada, and EBAA regulations require all corneal donors to undergo virologic testing for HIV, hepatitis B, and hepatitis C.^1^ The FDA prohibits any MSM donors who have been sexually active in the preceding 5 years, and Health Canada prohibits corneal donation by MSM donors who have been sexually active in the preceding 12 months; however, current HIV tests are reliable within days of viral exposure. All corneal donors in the United States and Canada now undergo HIV-1 enzyme-linked immunosorbent assay (ELISA) and HIV-2 ELISA, which are antibody tests with a window period of only 20 to 25 days (meaning that the test is reliable when performed at least 20-25 days after initial viral exposure).^17,18^ However, one advance has been the advent of HIV nucleic acid testing (NAT), which has a window period of 4 to 8 days.^8^ When performed little more than 1 week after a potential donor has been exposed to HIV, the sensitivity and specificity of HIV NAT is essentially 100%.^19^ The FDA and Health Canada also require testing for hepatitis B and hepatitis C, with FDA-licensed screening tests reliable within 20 to 22 days of exposure to hepatitis B and within 5 to 7 days of exposure to hepatitis C.^8,20^ The improved sensitivity and brief window periods of current virologic testing support reformulating the present MSM deferral policy.

There have been at least 10 reported cases in the literature of corneal transplants using tissue from donors who were found to be HIV-positive after surgery.^21,22,23,24^ None of the 10 corneal recipients from the HIV-positive donors contracted HIV, whereas all 12 recipients of solid organs from those same donors contracted the virus. Therefore, even in patients with a high enough HIV viral load for HIV to be transmissible via solid-organ transplant, the risk of HIV transmission via corneal transplant is low. Infectious disease transmission via corneal transplant is legally required to be reported in the United States, Canada, and the European Union; however, to our knowledge, no case of HIV transmission from a corneal transplant has been reported anywhere in the world.^25,26,27^ Even with strict social screening criteria, hundreds of donated corneas are discarded each year from otherwise eligible donors who are found to be HIV positive by antibody testing and NAT, so it is likely that some corneas are already being donated during the HIV window period without ever having led to a case of HIV transmission.^16^

One reason for the low transmissibility of HIV via corneal transplant is thought to be the cornea’s avascularity, which prevents the cornea from being a major reservoir of the virus.^28^ Studies analyzing the corneas of HIV-infected patients have consistently found that HIV is not present in most of the corneas of HIV-positive patients. One study^29^ analyzing 90 corneas recovered from HIV-positive patients found HIV antigen in only 6 of the 90 corneas. Another study^30^ performed HIV polymerase chain reaction on 22 corneas from patients who died of AIDS complications (presumably with high viral loads), and HIV was detected in only 4 of the 22 corneas. Thus, even if a corneal donor is able to donate eyes within a few days of being exposed to HIV (ie, too soon for serologic test results to return as positive), the odds of there already being an infectious level of HIV in the corneas within only a few days of initial HIV exposure are low.

Current FDA policy does not seem to be consistent regarding donation of corneas by MSM compared with donation of other biological material. Although the MSM population must be sexually abstinent for 5 years before corneal donation, blood donation requires only 3 months of abstinence by MSM donors in the United States.^31^ A prior lifetime ban on MSM blood donation was changed to a 1-year deferral in December 2015, when an FDA review concluded that current evidence no longer supported a lifetime ban on MSM donors.^32^ This policy was further amended on April 2, 2020, when the FDA announced that blood donors should be deferred for only 3 months after last MSM contact.^31^ No such reevaluation has occurred for the 5-year deferral period for corneal donation. Regarding solid-organ donation, there is no deferral period for MSM donors whatsoever, who are allowed to donate their hearts, lungs, kidneys, and livers without delay.^33,34^ This policy is despite the fact that HIV is well known to be transmissible via blood transfusion and solid-organ transplants but again has never been reported in corneal transplants.^28^

Compared with other potentially high-risk donors, MSM corneal donors are also subject to stricter screening criteria. For example, current FDA policy states that a heterosexual person who has been in a sexual relationship with someone known to be infected with HIV should be deferred for only 1 year after the last sexual contact with the infected individual, whereas a hypothetical monogamous MSM donor who has never been exposed to HIV would remain ineligible to donate his corneas for 5 years after his last sexual contact.^6^

The loss of 1558 to 3217 corneas a year is especially substantial when one considers the estimated 12.7 million people across the world in need of a corneal transplant, with only 1 cornea available for every 70 corneal transplants needed.^9^ Although eye banking infrastructure in the United States is able to meet local demand, most countries are not so fortunate. As recently as 2012, only the United States, Italy, and Sri Lanka were major exporters of surplus corneas.^9^ The rest of the world is forced to rely on a mix of wait lists, stricter visual thresholds for transplant eligibility, or importation of corneas from export countries. In many countries, such as Japan and Canada, the wait list for a corneal transplant can be several years.^35,36^ Public records in Argentina show that in February 2020 over 30% of the patients on the wait list for a corneal transplant had been waiting more than 3 years, with over 100 patients who had been waiting at least 7 years.^37^ Corneas imported from the United States, Italy, and Sri Lanka are an important means of addressing this unmet need across the world.^38^ With more than 25 000 corneas exported from the United States annually, the FDA’s policy banning corneal donation by sexually active MSM donors has deprived thousands of visually impaired people since 1994 from receiving vision-restoring surgery.^16^

The United States and Canada are outliers with respect to corneal donation by MSM donors. Many nations, such as Spain, Italy, Mexico, Chile, and Argentina, have no MSM deferral period for corneal donation.^39,40,41,42,43,44^ Their donor screening policies make no distinction between heterosexual and MSM donors. Of note, Italy exports more than 600 corneas a year, and eye banks in other nations are accepting such tissue even though Italy has no prohibition on MSM corneal donation.^9^ Current European Union regulations allow for corneal donation by the MSM population, although individual member nations are able to adopt their own stricter deferral criteria.^45^

Some nations continue to defer MSM corneal donors but for far shorter periods than 5 years or 12 months. For example, the United Kingdom allows MSM corneal donation after only 3 months of abstinence,^46^ whereas the Netherlands requires 4 months of abstinence for MSM corneal donors.^47^ Similarly, the consensus of French tissue banks is to defer MSM corneal donors for a period of 4 months after last MSM contact (Isabelle Martinache, Agence de la Biomédecine, email communication, March 23, 2020). Such deferral periods are timed based on the effectiveness of modern virologic testing, with a brief buffer of extra time out of caution. With HIV testing reliable within days of viral exposure and hepatitis testing reliable within a few weeks of exposure, the FDA and Health Canada could update their MSM deferral policy to a period of only 3 or 4 months, which would more than double the period needed for reliable virologic testing.

### Limitations

Our study has some limitations. It was limited by reliance on a nonvalidated survey as well as regional differences in the size of the MSM population. The fact that the survey incorporates data from eye banks supplying almost half of all corneas recovered in the United States and Canada lessens the consequences of selection bias, and the survey captured data from a wide variety of urban and rural eye banks across North America (Table 1). Our survey relied on data from institutions with varying systems of record keeping, with many of the 24 eye banks reporting that their MSM deferral data were incomplete because the level of detail in the documentation was technician dependent, and some MSM donors were simply labeled as high risk without a specific disqualifying factor documented in their records. The MSM deferrals that were labeled this way would have been missed by the present analysis, and the study’s retrospective nature prevented eye banks from ensuring that their technicians documented MSM status consistently. Furthermore, some eye banks reported that their referral partners are aware of donor eligibility criteria and do not always notify the eye bank when an MSM patient dies. Such patients would also have been missed by our analysis. These limitations suggest that our lower estimate of 1558 corneas may be an underestimate. However, the higher estimate of 3217 corneas was likely an overestimate because eye banks inherently rely on a social history provided by a donor’s next of kin, who are not always aware of donor MSM status. The actual number of donations prevented in 2018 because of the MSM deferral policy likely lies somewhere between the 2 estimates of 1558 and 3217 corneas.

## Conclusions

Armed with modern screening tests and the knowledge that corneal transplant is an unlikely means of HIV transmission, the 5-year and 12-month MSM deferral policies are no longer supported by current evidence. With millions of people across the world in need of corneal transplants, the 1558 to 3217 corneas discarded each year in the United States and Canada because of this policy could be used to address the shortage and safely restore vision to patients with corneal blindness worldwide. These findings suggest that the FDA and Health Canada should consider the example of other nations by shortening or eliminating their MSM deferral policies for corneal donation.
